# Local and Systemic Humoral Response to Autologous Lineage-Negative Cells Intrathecal Administration in ALS Patients

**DOI:** 10.3390/ijms21031070

**Published:** 2020-02-06

**Authors:** Bartłomiej Baumert, Anna Sobuś, Monika Gołąb-Janowska, Zofia Ulańczyk, Edyta Paczkowska, Karolina Łuczkowska, Alicja Zawiślak, Sławomir Milczarek, Bogumiła Osękowska, Agnieszka Meller, Karolina Machowska-Sempruch, Agnieszka Wełnicka, Krzysztof Safranow, Przemysław Nowacki, Bogusław Machaliński

**Affiliations:** 1 Department of General Pathology, Pomeranian Medical University, 70-111 Szczecin, Poland; bbaumert@pum.edu.pl (B.B.); ania.sobus@gmail.com (A.S.); z.litwinska@gmail.com (Z.U.); edyta.paczkowska@pum.edu.pl (E.P.); karolinaluczkowska58@gmail.com (K.Ł.); alicja.zawislak@pum.edu.pl (A.Z.); slawek.milczarek@gmail.com (S.M.); bogumilaosekowska@gmail.com (B.O.); 2 Department of Neurology, Pomeranian Medical University, 71-252 Szczecin, Poland; monikagj@op.pl (M.G.-J.); agoschorska@gmail.com (A.M.); karolinamachowska88@gmail.com (K.M.-S.); awelnicka@gmail.com (A.W.); przemyslaw.nowacki@pum.edu.pl (P.N.); 3 Department of Biochemistry and Medical Chemistry, Pomeranian Medical University, 70-111 Szczecin, Poland; chrissaf@mp.pl

**Keywords:** amyotrophic lateral sclerosis (ALS), lineage-negative cells, neurotrophins, gene microarrays, inflammatory response

## Abstract

Amyotrophic lateral sclerosis (ALS) remains a fatal disease with limited therapeutic options. Signaling via neurotrophins (NTs), neuroinflammation, and certain micro-RNAs are believed to play essential role in ALS pathogenesis. Lineage-negative stem/progenitor cells (Lin^−^) were obtained from bone marrow of 18 ALS patients and administered intrathecally. Clinical assessment was performed using ALS Functional Rating Scale (FRSr) and Norris scale. Protein concentrations were measured in plasma and cerebrospinal fluid (CSF) by multiplex fluorescent bead-based immunoassay. Gene expression in nucleated blood cells was assessed using gene microarray technique. Finally, miRNA expression was analyzed using qPCR in CSF and plasma samples. We observed a significant decrease of C-reactive protein (CRP) concentration in plasma on the seventh day from the application of cells. Gene array results revealed decreased expression of gene sets responsible for neutrophil activation. Further analysis revealed moderate negative correlation between CRP level in CSF and clinical outcome. Brain-derived neurotrophic factor (BDNF) concentrations in both plasma and CSF significantly correlated with the favorable clinical outcome. On a micro-RNA level, we observed significant increase of miR-16-5p expression one week after transplantation in both body fluids and significant increase of miR-206 expression in plasma. Administration of Lin^−^ cells may decrease inflammatory response and prevent neurodegeneration. However, these issues require further investigations.

## 1. Introduction

Amyotrophic lateral sclerosis (ALS) is a progressive, adult-onset neurodegenerative disease leading to gradual loss of motor neurons (MNs) and consequently to death in approximately 3–5 years after diagnosis, mostly due to respiratory failure [1]. It was first described in 1869 by Jean-Martin Charcot—one of the world’s pioneers of neurology [2]. His initial statements that the disease was never inherited and affected only motor neuron were found false in the 20th century when it was described that, in some patients, the cognitive impairment occurs together with muscle weakening, and in about 10% of cases, the disease affects members of the same family [3,4]. Still, the most frequent (about 90% of cases) form of ALS is the sporadic one (sALS) in which the underlying genetic mutation remains unknown [5]. In Europe, the incidence of ALS is estimated at 2–3/100,000 and is higher among men [6]. Based on the site of onset, the bulbar and the spinal-onset types of ALS are distinguished. Bulbar site of onset and older age at the time of diagnosis predict more rapid disease progression [3]. Among pathological mechanisms leading to MNs loss, the best-described ones are protein dysfunction, manifested as protein aggregation and misfolding, oxidative stress, glutamate excitotoxicity, mitochondrial abnormalities, and some environmental factors with cigarette smoking as the most consistent one [7,8,9,10,11]. In ALS, pathological changes affect multiple types of cells, including astrocytes, oligodendrocytes, Schwann cells, and microglia, but the earliest changes can be found in MNs and their synapses [12]. It is suggested that degeneration of these cells may be a consequence of an ongoing autoimmune process or related to increased inflammatory response [13,14]. 

Despite the intensive research up to date, there is no effective treatment that would allow significant inhibition or arrest of disease progression. Until 2017, riluzole—a neuroprotective drug that acts by blocking the glutamatergic neurotransmission in the central nervous system—was the only Food and Drug Administration (FDA) approved medication administered to patients with this fatal condition [15]. In May 2017, the FDA approved edaravone, an agent known for its antioxidant properties, for treatment in ALS patients [16]. Riluzole prolonged survival of patients by 2–3 months compared to placebo in clinical trials [17]. Edaravone seems to decrease disease progression, measured by revised ALS Functional Rating Scale (ALS-FRSr), when compared to the placebo group, but only in early-stage ALS patients [18]. In general, both available drugs show more profound effects when the disease is diagnosed in early stages. However, the time to make an ALS diagnosis is reported to range from 8 to 15 months [19], which often impedes the introduction of early treatment and reduces its effectiveness. Lack of effective treatment and incomplete understanding of the underlying pathology of ALS have turned researchers’ attention towards finding other therapeutic approaches. One of them is the application of stem and progenitor cells. Different populations of cells derived from various types of tissues have been used in ALS clinical trials. The general approach is not to replace motor neurons that undergo degeneration in the course of the disease but rather to benefit from stem cells’ (SCs) abilities to produce growth factors and affect inflammation and immune response through release of anti-inflammatory cytokines [20]. 

In our study, we focused on bone marrow-derived progenitors, lineage-negative (Lin^−^) cells that do not present on their surface cluster of differentiation antigens characteristic for mature hematopoietic cells [21]. In turn, this cell population is enriched in stem/progenitor cells (SPCs) expressing CD34 and CD133 antigens. Moreover, our studies on Lin^−^ cells isolated from umbilical cord blood revealed that chemokine receptor type 4 (CXCR4), which is involved in migration and homing of bone marrow SPCs, is also highly expressed on their surface [22]. We previously proved that Lin^−^ cells intrathecal administration is a safe and feasible method to apply in ALS patients [23]. This cell population is able to secrete neurotrophic factors under stress conditions, e.g., after incubation in serum-free media, which resembles low protein content in cerebrospinal fluid [22,24]. In vitro studies utilizing cord blood-derived Lin^−^ cells have shown that they can transdifferentiate into neuroglial progenitors [25]. Those properties make them an interesting candidate for studies aimed at delivery of trophic support to the central nervous system. 

The group of growth factors known as neurotrophins (NTs) is of particular interest when it comes to neurodegenerative diseases. They exert their actions via binding to transmembrane receptors of two classes—Trks and p75 [26]. They regulate neuronal growth, survival, plasticity, and function of neurons [27]. In animal models of motor neuron disease, brain-derived neurotrophic factor (BDNF) and neurotrophin-3 (NT-3) decreased MNs degeneration and contributed to increased lifespan of the studied animal group [28,29]. However, in clinical trials, the subcutaneous injections of recombinant BDNF have failed to prolong patient survival [30]. One of the characteristics of NTs that may impair their potential neuroprotective properties in humans is their short half-life and relatively big molecular size, which after subcutaneous injection may cause difficulties with crossing the blood–brain barrier [31]. 

Recently, much importance has been attributed to the role of neuroinflammation in neurodegenerative diseases. C-reactive protein (CRP) levels are seen as a potential biomarker in ALS [32]. CRP could be produced not only by the liver, as was originally thought, but also locally in the brain [33]. It seems that this production is enhanced in areas damaged by neurodegenerative processes, for example, in Alzheimer’s disease [34]. In ALS patients, CRP levels could correlate with neurologic functional impairment and survival [35]. In our previous study, we investigated the role of neuroinflammation in the course of ALS, including CRP and complement components, i.e., C3 and C4 [23]. In this study, we investigated CRP as a marker of inflammation at various time points after cells injection. Parallel, we aimed to assess changes in inflammatory processes activation on gene expression level using microarrays, as it was previously noted that ALS patients present with different numbers of leukocytes and their subpopulations compared to healthy control patients [36].

MicroRNAs are small, non-coding RNA fragments that act as epigenetic regulators of gene expression and have been proposed as candidates for biomarkers of several diseases, including neurodegenerative ones [37,38]. Among those related to regulation of processes associated with neuronal cells survival and function, we selected candidates that were previously described as involved in ALS occurrence and disease progression. miRNA-16-5p was suggested to exert neuroprotective effects in studies on murine models of neurodegenerative diseases [39,40]; however, it still lacks deeper investigation in ALS patients in this context. A second analyzed miRNA was miR-206, which was proposed as a marker for ALS [41]. It was reported that miR-206 promotes regeneration of neuromuscular synapses, and its deficiency accelerates ALS progression in a murine model [42]. miR-9 was shown to promote proliferation of neuronal progenitor cells, and it proved to be richly represented in the brain [43]. The last analyzed miRNA was miR-let-7f-5p, expression of which decreases in plasma of ALS patients [44].

Based on our previous positive results with cell administration in ALS patients, in this study, we aimed to further explore this field. We focused on neurotrophins for their neuroprotective action and on CRP as an indicator of inflammatory response. Our goal was also to assess the correlation between functional outcome and concentration of these factors in ALS patients after intrathecal Lin^−^ cells administration in various time points. Furthermore, we sought to investigate influence of Lin^−^ cells administration on regulatory pathways on a micro-RNA level as well as on the intensity of inflammatory response.

## 2. Results

### 2.1. Clinical Assessment

We assessed the functional condition of enrolled patients based of two scales—the Norris scale and the ALS-FRSr. Assessment was performed zero, three, five, seven, and 28 days after administration of autologous Lin^−^ cells isolated from bone marrow. Two of the enrolled patients did not attend the examination on the 28th day. After analysis of the collected data, in one patient, the Norris scale score increased slightly in the initial days from zero to five (from 103 to 104 points), while in the cases of other patients, the score in the first week after Lin^−^ cells injection remained unaffected. Therefore, we analyzed changes in the functional outcome only based on the score shift between days zero and 28. Based on such assessment, we observed that, in this time period of post cell application, the results in the Norris scale did not change by more than one point in nine individuals, increased by at least two points in three patients, and decreased by more than two points in four patients. When it came to assessment using ALS-FRSr, the number of patients whose condition remained stable, improved, or deteriorated were the same as with analysis using the Norris scale. Described changes are presented in Table 1. 

### 2.2. Concentration of CRP and Selected NTs

Using Luminex multiplex assay, we investigated concentrations of CRP and three selected neurotrophic factors—BDNF, nerve growth factor beta (NGF beta), and NT-3. Blood plasma samples were collected on day zero and on the third, the fifth, and the seventh days post cell injection. 

Plasma concentrations of CRP were lower one week after cells administration than on day zero. Using the Friedman test, we observed that the decrease was statistically significant (*p* = 0.032). The obtained data were then subjected to further statistical analysis using paired Wilcoxon test to compare changes between certain time-points. We observed a statistically significant difference in CRP concentration between results obtained on day zero and day seven after Lin^−^ cells administration (*p* = 0.006) and between the fifth and the seventh day (*p* = 0.006). 

**Figure 1:**
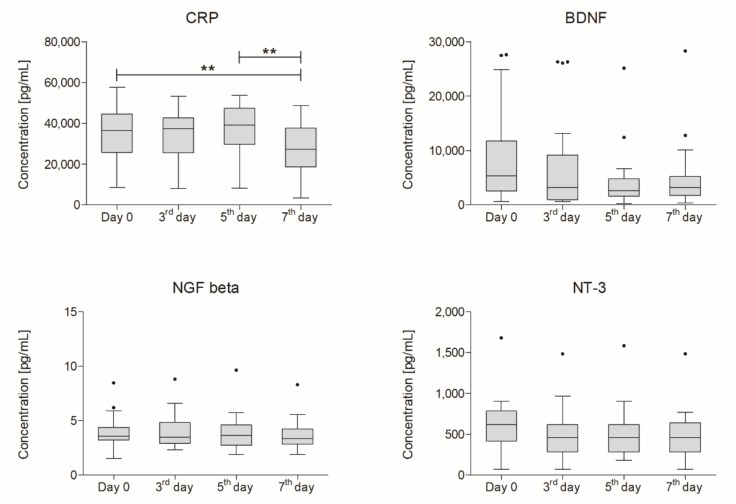
Boxplot representing concentrations of analyzed factors in plasma (pg/mL) measured on days zero, three, five, and seven after intrathecal administration of bone marrow-derived lineage-negative cells (*n* = 18). Significance of the changes between analyzed timepoints was assessed using Wilcoxon signed-rank test. Whiskers range from 10th to 90th percentile. Horizontal line inside the box indicates the median. Level of significance-** *p* < 0.01. CRP: C-reactive protein; BDNF: brain-derived neurotrophic factor; NGF: nerve growth factor beta; NT-3: neurotrophin-3.

Analysis of three selected neurotrophic factor concentrations also revealed a decrease in their levels. None of those, however, were significant. Changes in plasma concentrations of selected factors are presented in Figure 1.

**Figure 2:**
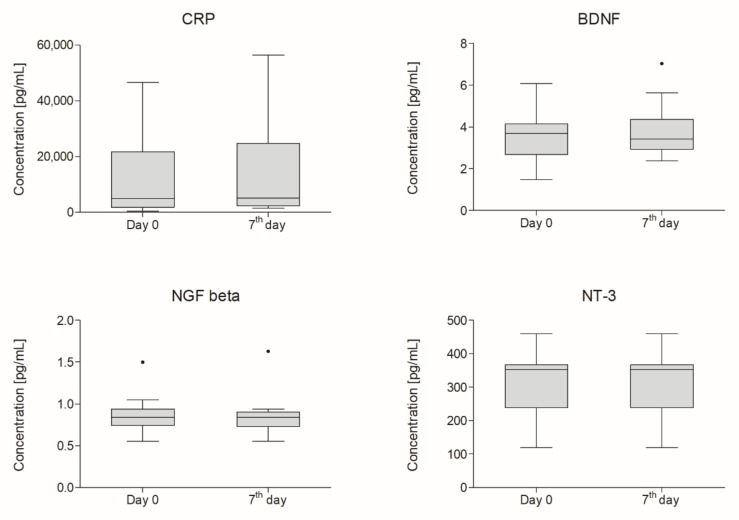
Boxplot of cerebrospinal fluid (CSF) concentrations of analyzed factors (pg/mL) measured on day zero and a week after intrathecal application of lineage-negative cells (*n* = 18). Whiskers from 10th to 90th percentile. Horizontal line inside the box indicates the median.

CSF samples were collected via lumbar puncture before the injection of Lin^−^ cells suspension (day zero) and seven days later. CRP level and BDNF concentration in CSF increased, as opposed to changes observed in blood plasma. We did not observe any statistically significant differences between concentrations of analyzed factors on day zero vs. day seven. Obtained data are presented in Figure 2. 

### 2.3. Correlation Between Functional Outcome and Concentration of CRP and NTs

Correlations between analyzed parameters were evaluated using Spearman’s rank correlation testing. We compared changes, calculated as the difference between a given timepoint and day zero, and checked if there was a correlation with a patient’s functional outcome fluctuation (day 28 vs. day zero) according to both ALS assessment scales. We did not observe any correlations between analyzed parameters and sex. Therefore, both sexes were analyzed together. 

Results of correlation analysis between changes in CSF concentrations of analyzed factors and changes in a patient’s functional outcome are presented in Table 2. Analysis of the correlation between concentrations of assessed proteins in plasma and the ALS-FRSr and the Norris scale results are attached as Supplementary Materials File 1. It is worth stressing that a statistically significant moderate negative correlation between CRP level in CSF and clinical outcome was observed (*p* = 0.009). From the analyzed NTs, only BDNF concentrations in both plasma and CSF revealed significant negative and positive correlations with the clinical outcome, respectively (*p* = 0.019 and *p* = 0.033).

The number of harvested and isolated mononuclear and Lin^−^ cells in ALS patients varied individually. Analysis revealed a negative correlation between the numbers of Lin^−^ cells administered to the patient and CRP concentrations in CSF. The higher the number of isolated and injected Lin^−^ cells was, the lower the concentration of CRP in CSF was one week later (*r_s_* = −0.494, *p* = 0.04). Moreover, higher initial CRP levels in this body fluid were correlated with lower numbers of cells harvested on day zero (*r_s_* = −0.648, *p* = 0.004). However, when it came to correlation between cells count and functional outcome and concentrations of other measured factors, we did not observe any statistically significant results.

### 2.4. Gene Microarray Analysis

**Figure 3:**
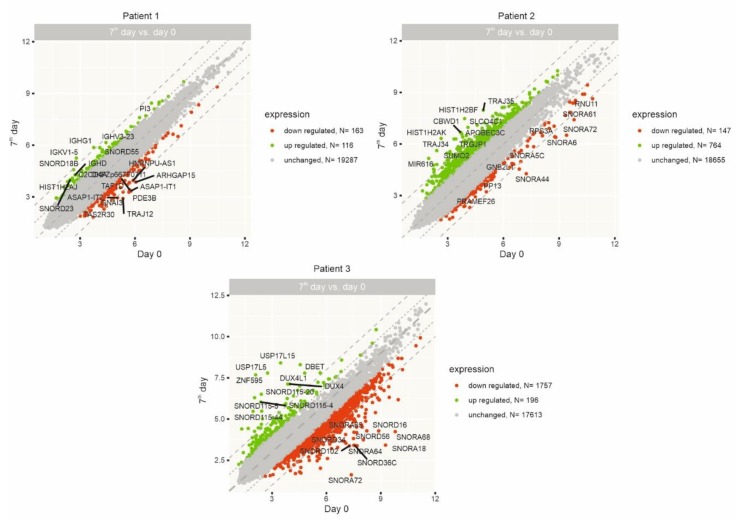
Scatter plots representing the most up- (green) and down-regulated (red) genes on the seventh day after Lin^−^ cells injection in comparison to day zero for each individually analyzed patient.

Gene expression was examined in samples obtained from three randomly chosen patients. The group consisted of two males and one female aged 61, 60, and 57. All patients presented with a spinal type of disease onset. Detailed characteristics of clinical parameters, numbers of isolated and administered cells, as well as functional results for those three patients are included in Supplementary Materials File 2. Gene microarray was used to assess gene expression in RNA isolated from peripheral blood nucleated cells, as we wanted to examine if intrathecal application of Lin^−^ cells affects response on a systemic level. Blood samples for RNA analysis were collected before the injection of Lin^−^ cells (day zero) and one week later (day seven). Genes whose expression changed significantly (at least a two-fold difference) on the seventh day in comparison to day zero for each patient are presented on scatter plots in Figure 3.

In addition, we enlisted 10 of the most up- and downregulated genes for each patient individually, which are attached as Supplementary Materials File 3. The most represented group of genes in the set of highly changed ones are those encoding small nucleolar RNAs (snoRNAa), which are responsible for methylation (class C/D) or pseudouridylation (class H/ACA) of ribosomal RNA [45]. They play an important role in maturation of rRNA and regulate mRNA splicing and editing, but there is also evidence of their involvement in other processes and cellular pathways, including those related to p53 tumor suppressor or phosphoinositide 3-kinase [46]. 

**Figure 4:**
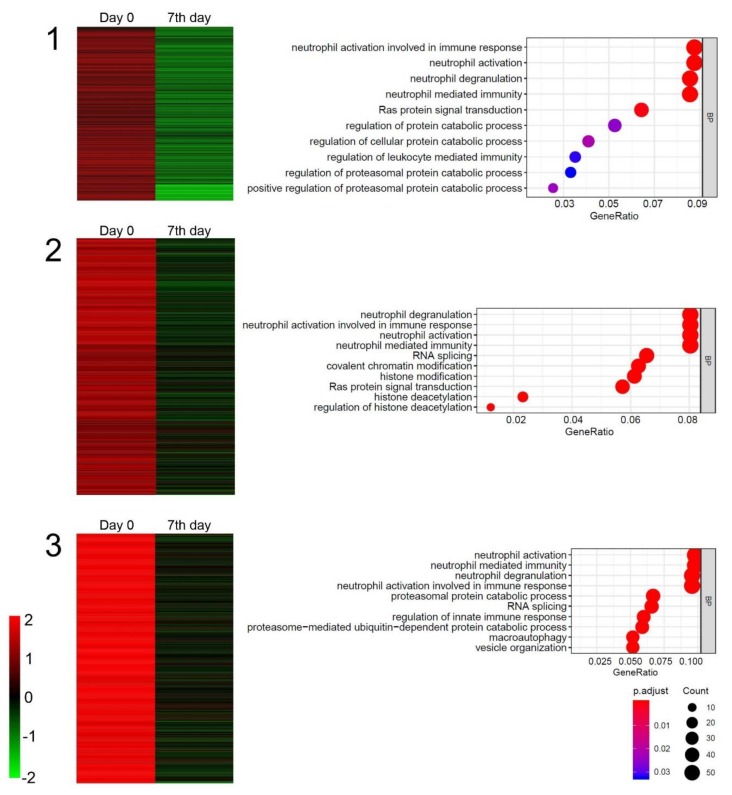
Heatmap presentation of cluster gene expression analysis using the K-means approach on day zero and day seven after Lin^−^ cells administration in peripheral blood nucleated cells obtained from three analyzed sporadic amyotrophic lateral sclerosis (sALS) patients (**1**–**3**). Bubble plots represent gene assignment to relevant GO ontological groups.

In the next step of analysis, we used the K-means clustering approach to identify differentially expressed genes (DEGs). We were particularly interested in those that behaved similarly in all analyzed patients in both time points. Genes belonging to each cluster were assigned to relevant gene ontology terms (GO) from the GO BP Direct Database. For all patients, we discovered decreased expression of a gene cluster that contained genes responsible for neutrophil mediated immunity, neutrophil degranulation, neutrophil activation, and neutrophil activation involved in immune response. Expression change of all genes from this cluster is presented in Figure 4. Additionally, the most represented GO terms from this cluster are presented as a bubble plot for each patient individually. A detailed list of genes from the presented cluster together with scaled and centered expression values and a correlation score is attached in Supplementary Materials File 4.

### 2.5. miRNA Expression Analysis

Expression levels of selected miRNAs in CSF and plasma of ALS patients were analyzed using q-RT-PCR in two timepoints, on the day of Lin^−^ cells administration and seven days after the procedure. Expression was normalized to exogenous spike-in *Caenorhabditis elegans* miR-39. We observed an increase in expression of miR-16-5p, miR-206, and miR-let-7f-5p in blood plasma on the seventh day after injection of Lin^−^ cells. In the case of miR-16-5p, the increase was statistically significant (*p* = 0.043). 

**Figure 5:**
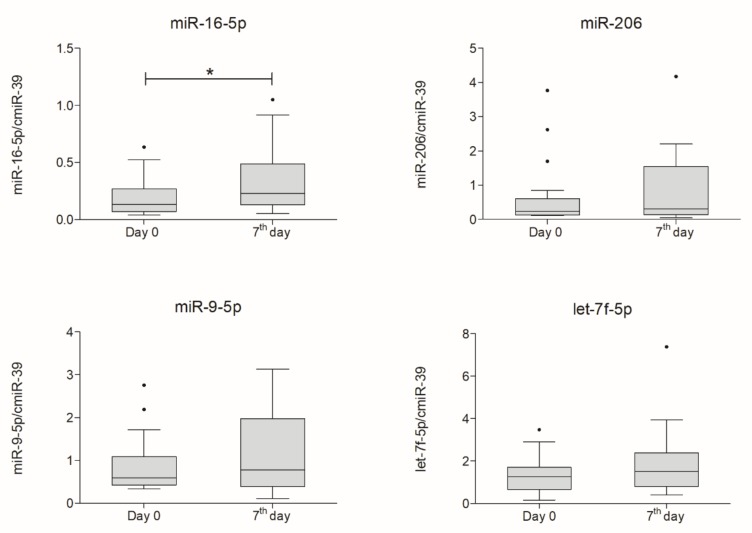
Boxplot representing plasma expression of analyzed miRNAs measured on day zero and a week after intrathecal lineage-negative cells application normalized to expression of synthetic cmiR-39 (*n* = 18). Statistical analysis was performed using Wilcoxon signed-rank test. Whiskers range from 10th to 90th percentile. Horizontal line inside the box indicates the median. Level of significance-* *p* < 0.05.

**Figure 6:**
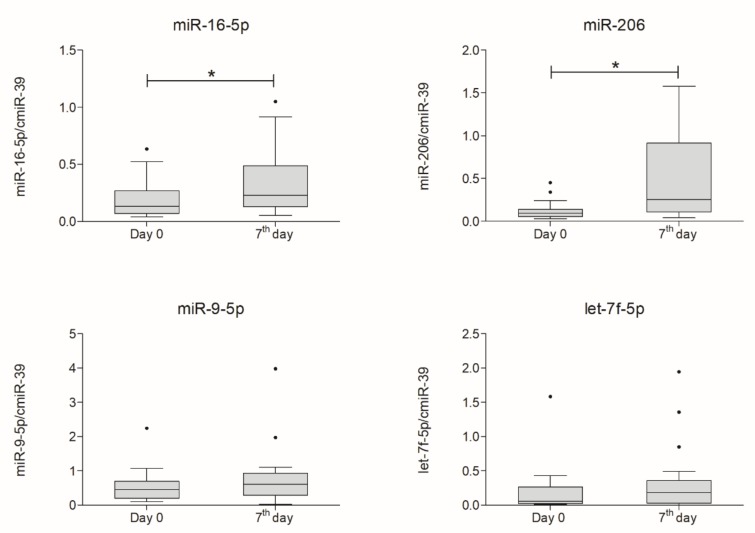
Boxplot of expression of analyzed miRNAs in cerebrospinal fluid measured on day zero and a week after intrathecal lineage-negative cells application (*n* = 18). Statistical analysis was performed using Wilcoxon signed-rank test. Whiskers from 10th to 90th percentile. Horizontal line inside the box indicates the median. Level of significance-* *p* < 0.05.

CSF miRNA expression analysis revealed a significant increase in miR-16-5p (*p* = 0.025) and miR-206 (*p* = 0.023). Expression of miR-9-5p and miR-let-7f-5p increased as well; however, changes were not significant. Results of miRNA expression in blood plasma and CSF are presented in Figure 5 and Figure 6, respectively.

## 3. Discussion

Pathophysiological processes that occur in the course of ALS are complex and likely influenced by a plethora of factors, including interaction between genes, environmental factors, and disturbed molecular pathways [47]. Unclear mechanisms that underlie neurodegenerative processes in ALS make drug development difficult. We previously demonstrated that intrathecal injection of autologous bone marrow-derived Lin^−^ cells is a safe and feasible method aimed at trophic provision in ALS patients [23]. In this study, we focused on neurotrophins and CRP, because they could indicate ongoing neuroprotection or decreasing inflammation as an effect of Lin^−^ cell application. We studied these factors and their concentration after Lin^−^ cells intrathecal administration in both CSF and plasma in order to examine both local and systemic responses. Our main goal was to assess the correlation between the functional outcome of the disease and the concentration of these factors in ALS patients after Lin^−^ cells administration. 

Neurotrophins have long been characterized as major governors of survival, maintenance, and regeneration of neurons to a degree that they are actually considered valuable treatment options for neurodegenerative diseases, ALS included [48]. In our study, concentrations of neurotrophins (BDNF, NGF, NT-3) did not substantially change after Lin^−^ cells administration in both CSF and in plasma. This may have been due to the generally short half-life of these molecules [49,50]. Perhaps, repeated cells injections could overcome this and be beneficial for a longer period of time, but this requires further investigation. From all tested neurotrophins, BDNF was the only one for which any correlation with the clinical outcome was observed. BDNF plasma concentration decreased on the fifth day and showed a moderate negative correlation with ALS-FRSr score, whereas BDNF concentration increased in CSF seven days after the cell injection and showed a moderate positive correlation with ALS-FRSr score. Several studies have described the involvement of the Superoxide Dismutase 1 (*SOD1*) gene, in which mutations account for 20% of familial ALS in the expression of BDNF [51]. Although we did not study familial ALS here, this SOD1-BDNF relationship suggests a close involvement of BDNF in the pathophysiology of ALS. In 2006, Bemelmans et al. described neuroprotective effects of BDNF in an excitotoxicity model [52]. More recently, Shruthi et al. showed that BDNF has a preventive role in neurodegenerative processes in an ALS in vitro model [53]. Schiaffino et al. further explored this idea with their studies, in which they recently proved that BDNF is down-regulated in the spinal cord of SOD1^G93A^-mice, while epigenetic drugs could enhance BDNF production in this model [54]. The discrepancy between plasma and CSF levels of BDNF in our study has several possible reasons. Firstly, neurothrophin plasma level could influence the CSF level results [26]. Secondly, the trend to a decrease in BDNF plasma concentration in time correlating with a better clinical outcome could suggest BDNF mobilization from blood to the locally damaged neurons. This hypothesis also corresponds with a significant moderate positive correlation of elevated CSF BDNF levels with a better ALS-FRSr score. However, in our study, the presumable BDNF mobilization was not reflected in its significant concentration increase in CSF. Interestingly, recent results by Tremolizzo et al. point out an opposite correlation of serum BDNF with clinical outcome compared to our observation [55], though it is unclear whether the type of sample (plasma vs. serum) could play a role here. The varying levels of BDNF in circulation of patients with neurodegenerative diseases once again support the idea of intrathecal administration of cells secreting BDNF so that it can act in the site of the injury. 

CRP has been described as a biomarker of the inflammatory response with a significant prognostic value in a vast number of tumors as well as rheumatic and cardiovascular diseases [56]. In our study, we observed a moderate negative correlation of decreasing CRP levels in CSF with clinical outcome in the patients. On the other hand, in plasma, CRP levels slightly peaked on day five and decreased on day seven, and the difference was statistically significant. Keizman et al. previously correlated CRP levels with neurological disability in ALS, though the study was performed on a small number of patients [57]. In a large case-control study from 2011, no association was found between CRP level and ALS [58]. However, a more recent study by Nagel et al. on 289 ALS patients and 506 controls showed a moderate inverse correlation of CRP with ALS-FRSr, which is in concordance with our results [59]. Also in 2017, another study on 394 ALS patients confirmed the CRP-ALS-FRSr correlation [60]. Not only the aforementioned clinical trials but also several other studies support the hypothesis that neuroinflammation is a poor prognostic factor that worsens with disease progression, just as degeneration progresses. In fact, Lu et al. established that IL-6 is associated with CRP levels and is the only marker that shows an increase in expression toward the end-stage of ALS [61]. However, these correlations were appropriate for serum CRP concentrations. As suggested previously, CRP in the peripheral blood may be a reflection of the local inflammation in the central nervous system [60]. In our case, only CRP in CSF was negatively correlated with ALS-FRSr (*p* = 0.009), and no significant correlation was observed in the case of plasma CRP. The gradual decrease of plasma CRP together with the inverse correlation of its CSF concentration with clinical outcome could potentially suggest beneficial effects of applicated cells—as CRP is decreasing, the clinical outcome is improving. Since the majority of studies on neuroinflammation in patients with neurodegenerative diseases have been conducted on peripheral blood samples rather than CSF, our results on CRP in CSF could potentially bring us closer to the core of ALS pathophysiology. To the best of our knowledge, this is the first study on CRP levels in CSF and their correlation with clinical outcome in ALS patients. Whether local neuroinflammation and CRP production could be direct prognostic factors or targets for therapy requires further studies, but our results seem promising. However, negative correlation between CRP in CSF and clinical outcome without the evident changes of CRP concentration in CSF and, at the same time, the lack of plasma CRP ALS-FRSr correlation with the evident changes in plasma CRP at different time points require further investigation. We also observed a negative correlation between the number of Lin^−^ cells administered and CRP concentrations in CSF seven days post injection. Those results stay in line with our other findings, which prove a decreased level of inflammatory response after administration of lineage-negative cells in ALS patients. Moreover, higher initial CRP levels in CSF correlated with lower numbers of cells harvested, which may show the implication of inflammatory response level on availability of lineage-negative cells pool.

Neuroinflammation is widely accepted as one of the factors contributing to degeneration of neural cells in the central nervous system of ALS individuals. Neutrophils are the most abundant group of leukocytes that take part in both the innate immune response and the regulation of inflammatory processes [62]. Trias et al. showed that mast cells together with neutrophils are abundant in motor neurons of symptomatic SOD1^G93A^ rats and that prevention of their infiltration is correlated with decreased demyelination and muscle fiber loss [63]. In our study, the analysis of gene expression revealed that genes encoding factors responsible for initiation of neutrophil activation expressed significantly lower levels on seventh day after intrathecal administration of Lin^−^ cells in comparison to a timepoint before injection. These results remain in concordance with decreased levels of CRP in plasma on the seventh day after cell application and suggest that an intrathecal route of administration results in systemic effects. However, a longer observation time is needed to more precisely determine time frames for neutrophil-related genes expression attenuation. 

miRNAs have been proposed as promising biomarkers for diagnosis and monitoring progression of various diseases [64], including ALS [37,65]. They have been discovered in many types of body fluids, including plasma, CSF, tears, saliva, colostrum, and peritoneal fluid [66]. In recent years, it was proposed that CSF miRNA in particular may indicate presence of brain tumors or neurological diseases, as CSF bathes and remains in direct contact with the central nervous system. Moreover, miRNAs are relatively stable, and they are less susceptible to degradation, even after storage in −20 °C, compared to longer RNA fragments [67]. Up to date, there is no standardized protocol for miRNA expression analysis. The approach used for unification of input miRNAs is based on total RNA quantification. However, in RNA-poor sample types, it is often impossible to precisely assess its concentration using spectrophotometric measurements. Solutions that enable normalization of miRNA content between analyzed samples are then the sample volume standardization and the addition of synthetic spike-in miRNA, which is used for normalization of expression results. In our study, we used 400 µL of both blood plasma and CSF sample types. Before the isolation, spike-in miR-39 from *C. elegans* was added to each sample. In our previous study, we found consistent expression of several tested miRNAs in CSF and plasma of ALS patients [23]. miRNA-16-5p downregulation was shown in ALS patients compared to controls [68,69] and in a murine model of early-onset Alzheimer’s disease (AD) [39]. Liu et al. suggested a neuroprotective role of miRNA-16-5p since, upon administration of miR-16-5p into the brains of AD mice, amyloid precursor protein levels were reduced [39]. More evidence of miRNA-16-5p’s neuroprotective role was brought by Majer et al. in their study on prion-infected mice, which presented an upregulation of miR-16-5p in early pre-clinical disease, with expression levels reducing as the disease progressed [40]. The observed upregulation of miRNA-16-5p expression in plasma and CSF of ALS patients on the seventh day after Lin^−^ cell administration in our study could reflect a neuroprotective response to cell therapy but could also be related to disease progression. The second miRNA chosen for this study, miRNA-206, belongs to a group of “myo-miRs”, miRNAs specifically expressed in striated muscle and involved in muscle proliferation and regeneration [70]. High expression levels of miRNA-206 were found in the murine model of ALS, and its downregulation has been linked with faster disease progression [42]. Our results are in agreement with previous research indicating elevated miRNA-206 in ALS patients [38,41,71,72], though we only observed this elevation in CSF, not plasma. It still remains unclear whether aberrant expression of miRNA-206 is the cause or the result of ALS. In the case of two other mi-RNAs analyzed in our study, we did not observe any significant changes in their expression levels. However, in CSF, we saw an increase in expression of miRNA-9, which has been under investigation as a regulator of neural cells development [73,74]. Haramati et al. reported that this micro-RNA is down-regulated in the genetic model of spinal muscular atrophy, which suggests its role in the development of neurodegeneration [75]; therefore, its increase in the environment of CNS may have beneficial influence on neural cells. Using high-throughput next-generation sequencing, Liguori et al. showed that miR-let-7f-5p is down-regulated in peripheral blood of sALS patients [69]. Similar findings were reported by Takahashi et al., this time after analysis of ALS patients’ plasma samples. The study of the aforementioned group stated also a negative correlation between let-7f-5p level and ALS-FRS-R bulbar paralysis score [44]. 

### Potential Study Limitations

Although our study provided valuable results, it did have some drawbacks. The first is the heterogeneity of the studied group; large deviation was observed in terms of age at disease onset, symptom duration, and number of collected and administered Lin^−^ cells. However, this diversity of the study group is actually a good depiction of ALS heterogeneity. Moreover, the experiment was carried out without the control group, which limited the possibility to draw direct conclusions of the effects of Lin^−^ cells application. In the case of a fatal neurodegenerative disease such as ALS, it seems rather unethical to recruit patients without offering them a chance for potentially beneficial intervention. However, since this is still a preliminary study of such cell administration, we must be cautious when interpreting the obtained data. Taking into consideration the complexity of ALS pathophysiology, it is still unclear whether the described correlations favor the Lin^−^ cells application as a beneficial treatment strategy for ALS or if they reflect other unknown factors.

## 4. Materials and Methods

### 4.1. Patients

In total, 18 patients, 9 male and 9 female, between 28 and 65 (53.1 ± 11.1) with sporadic ALS diagnosed according to El Escorial Revised Criteria [76] were enrolled in the study after it was approved by the Ethics Committee of the Pomeranian Medical University in Szczecin (approval code: KB-0012/06/10; 25 January 2010), and all patients declared written informed consent. Patients older than 65 and with any evidence of comorbidities were excluded from the study. Riluzole treatment was continued during the whole course of the study. Detailed characteristics of the studied group are presented in Table 3. The trial (international number: NCT02193893) was performed in accordance with the Declaration of Helsinki. 

### 4.2. Clinical Assessment

To assess the disease progression on a functional level, we used the revised ALS Functional Rating Scale and the Norris scale. ALS-FRSr is based on a questionnaire that can be divided into four main domains: bulbar function, fine motor function, gross motor function, and respiratory function, with a total score ranging from 0 to 48 points. ALS-FRSr is the most widely used functional ALS assessment scale, as it enables the evaluation of physical functions in daily living activities [77]. Norris scale focuses on limb and bulbar functions and is composed of 34 items [78]. Patients were evaluated before the administration of Lin^−^ cells and 3, 5, 7, and 28 days after application.

### 4.3. Bone Marrow Collection and Lin^−^ Cells Isolation

Bone marrow (BM) was aspirated on day 0 from the posterior iliac crest. On average, the total volume of BM with anticoagulant solution (saline solution and heparin) of 109.8 mL was collected. In a controlled environment of a closed incubation system, bone marrow was diluted 1:1 with phosphate-buffered saline (PBS) and layered on Lymphocyte Separation Medium (MP Biomedicals, Santa Ana, CA, USA). Obtained mononuclear cells suspension was then subjected to negative immunomagnetic isolation of Lin^−^ cells (Miltenyi Biotec, Auburn, AL, USA) following manufacturer’s protocol according to the GMP conditions. After isolation, cells were suspended in 2 mL of PBS. 

### 4.4. Intrathecal Injection of Lin^−^ Cell Suspension

Total isolated cells were injected; therefore, the number of administered cells differed between patients (5.5 ± 4.4 × 10^6^). Cells were injected into the subarachnoid space via the lumbar puncture (between L3/L4 or L4/L5). After injection, patients were recommended to maintain supine position for at least 24 h. No adverse or severe adverse events were reported.

### 4.5. Sample Collection

To assess the concentration of selected neurotrophic factors, plasma samples were collected on the day of bone marrow collection (day 0) as well as 3, 5, and 7 days after injection. In our previous work, we established that fluctuations in NTs levels after administration of Lin^−^ cells decreased after 7th day post-injection [23]. Therefore, the plasma samples were collected only up to this certain point. Cerebrospinal fluid (CSF) was collected in the time of Lin^−^ cells injection before cell administration (day 0) and after one week. Both sample types, plasma and CSF, were centrifuged to separate cellular content, and supernatant was stored in 500 µL aliquots in −80 °C until further analysis. 

### 4.6. Luminex Multiplex Assay

Concentrations of selected neurotrophic factors (NT-3, NGF, BDNF) and CRP were assessed in CSF and plasma using multiplex fluorescent bead-based immunoassays (Luminex Corporation, TX, USA). Assays were performed according to manufacturer’s protocol, as described previously [79]. Final concentration of the analyzed protein was assessed based on the averaged readings (average %CV = 6.8) obtained from both duplicates of a given sample in relation to four (one for each of the analytes) 6-point standard curves showing median fluorescence intensity. Before each run, the Luminex 200 analyzer was calibrated, and the performance verification was performed according to manufacturer’s instructions.

### 4.7. Gene Chip Microarray

Randomly, we chose samples collected from 3 sALS patients for gene expression analysis. The goal of the analysis was to determine gene expression changes after transplantation of lineage-negative cells with particular emphasis on genes regulating immune processes implicated in survival and differentiation of neural cells. Total RNA was isolated from peripheral blood mononuclear cells with PARIS Kit (Thermo Fisher Scientific, MA, USA) before cells transplantation (day 0) and one week later. Obtained RNA was then used to generate sense-strand cDNA, which was later fragmented, labeled, and hybridized onto a Human Gene 2.1 ST Gene Chip (Affymetrix, CA, USA). Affymetrix GeneAtlas™ System was used to perform all subsequent reaction steps: hybridization, washing, staining, and scanning of the array strip. Quality of performed reactions was checked using Affymetrix GeneAtlas Operating Software according to provided criteria. Output CEL files were analyzed using BioConductor software, which is based on R programming language. “affy” package was implemented to perform background correction, normalization, and summation of raw data. Annotations for biological processes were obtained from “oligo” package in BioConductor software. As reference data for expression changes analysis, we chose results obtained from samples collected before stem cells transplantation, as the main goal of the study was to assess the molecular changes caused by cells injection. We chose genes whose expression fold difference was higher than |2| as significantly changed. In order to identify the subgroups of co-regulated DEGs that behave similarly in all of analyzed comparisons, we used the K-means clustering approach [80]. Clustering was performed on the mean expression values of DEGs from each experimental group. Number of clusters was determined using sum of squared error (SSE) estimation. Then, the mean expression values were scaled and centered. Data were then subjected to the K-means clustering procedure and the next correlation of each gene from the clusters with clusters centroid (cluster core) values calculated. Genes below 0.8 correlation score were filtered out. Obtained results were visualized as a heatmap using “pheatmap” library. Genes belonging to individual clusters were subjected to functional annotation analysis using the DAVID (Database for Annotation, Visualization, and Integrated Discovery) bioinformatics tool [81]. Gene symbols from each cluster were uploaded to DAVID by the “RDAVIDWebService” BioConductor library, where they were assigned to relevant GO terms from the GO BP Direct database [82]. 

### 4.8. miRNA Expression Analysis

miRNA was isolated from blood plasma and CSF samples collected before injection of Lin^−^ cells and one week later (day 0 and day 7). Isolation of miRNA from collected samples was performed using NucleoSpin miRNA Plasma (Macherey-Nagel, Germany) according to the protocol. Then, 400 µL of each sample were used for isolation, and miRNA was eluted with 20 µL of nuclease-free water. Before isolation, samples were spiked-in with 25 fmol of synthetic *C. elegans* miR-39 (5′–TCACCGGGTGTAAATCAGCTTG–3′). Until first strand cDNA synthesis, samples were stored in −80 °C. Reverse transcription was performed with qScript microRNA cDNA Synthesis Kit (QuantaBio, Beverly, MA, USA). For reverse transcription, we used 4 µL of each miRNA sample. Then, 5 µL of each cDNA sample were used for preamplification PCR for 5 cycles with primers specific for analyzed miRNAs and universal miRNA primer provided by vendor (QuantaBio, Beverly, MA, USA) as described previously by Jazwa et al. [83]. Expression analysis of selected miRNA was carried out using Bio-Rad CFX96 thermal cycler (Bio-Rad, Hercules, CA, USA). The reaction mix contained cDNA, iQ SYBR Green Supermix (Bio-Rad, Hercules, CA, USA), a primer specific for analyzed miRNA, and universal primer provided with the first strand cDNA synthesis kit (QuantaBio, Beverly, MA, USA). Relative expression of analyzed miRNAs was normalized to spike-in control miRNA and calculated as 2^−ΔΔCT^.

### 4.9. Statistical Analysis

Statistical calculations of data obtained using Luminex and qPCR techniques as well as data characterizing the studied group of patients were performed using Statistica 13. Normal data distribution was tested with Shapiro–Wilk test. Friedman test was used to analyze changes of assessed parameters on both a clinical and a molecular level. Non-parametric Wilcoxon-signed rank test was utilized to compare mean values between two time points. Finally, to assess correlation rank between measured variables, we implemented Spearman’s rank order correlation testing. *p*-value < 0.05 was considered significant. 

## 5. Conclusions

In this preliminary study, we evaluated correlations between clinical outcome in ALS patients after Lin^−^ cells intrathecal administration and plasma/CSF levels of neurotrophins and CRP at various time points after cells injection. A significant decrease of CRP concentration in plasma was observed on the seventh day from the application of cells. Further analysis revealed moderate negative correlation between CRP level in CSF and clinical outcome. BDNF concentrations in both plasma and CSF significantly correlated with the favorable clinical outcome. Gene array results revealed decreased expression of gene sets responsible for neutrophil activation. On a micro-RNA level, a significant increase of miR-16-5p expression one week after transplantation in both body fluids and a significant increase of miR-206 expression in plasma were observed. Altogether, the results suggest the direct cause and effect relationship between autologous Lin^−^ cells administration and the organism’s systemic response. This study provides a closer look at local and systemic humoral response to autologous Lin^−^ cells application on protein, molecular, and epigenetic levels. It is worth mentioning that data presented in this paper represent the first phase of an ongoing study in which we are planning to assess the effects of a triple subsequent administration of Lin^−^ cells in ALS patients on a molecular and a functional level.

## Figures and Tables

**Table 1:** Results of clinical assessment using amyotrophic lateral sclerosis (ALS) Functional Rating Scale (FRSr) and Norris scale 28 days after administration of lineage-negative cells.

Score Fluctuations	Norris Scale	ALS-FRSr
Stable (score in day 28 the same as in day 0 or ± 1 point)	*n* = 9	*n* = 9
Increase (by at least 2 points)	*n* = 3 (mean = +11 ± 8.66)	*n* = 3 (mean = +4 ± 1)
Decrease (by at least 2 points)	*n* = 4 (mean = −6.75 ± 5.5)	*n* = 4 (mean = −4.5 ± 2.08)

**Table 2:** Correlations analysis between CSF concentration of selected factors and clinical outcome measured using ALS-FRSr and Norris scale. rs: Spearman’s correlation coefficient. *p* < 0.05 is highlighted with bold font.

CSF Concentrations		ALS-FRSr day 28 vs. 0 (Valid Calculations *n* = 16)		Norris day 28 vs. 0 (Valid Calculations *n* = 16)	
		*r_s_*	*p* Value	*r_s_*	*p* Value
**CRP**	day 7 vs. 0	−0.631	**0.009**	−0.483	0.080
**BDNF**	day 7 vs. 0	0.534	**0.033**	0.360	0.207
**NGF beta**	day 7 vs. 0	0.098	0.719	−0.288	0.318
**NT-3**	day 7 vs. 0	0.046	0.866	0.099	0.737

**Table 3:** Characteristic of studied group with ALS-FRSr and Norris scale results. Tx: transplantation.

Parameter		ALS Patients (*n* = 18)
**Age (mean ± SD, years)**		53.11 ± 11.14
Sex (male/female)		9/9
Disease onset (bulbar/spinal)		2/16
Age at disease onset (mean ± SD, years)		50.00 ± 12.95
Symptom duration (mean ± SD, months)		37.39 ± 34.93
Bone marrow volume (mean ± SD, mL)		109.78 ± 21.37
Number of mononuclear cells (mean ± SD)		200 x 10^6^ ± 95.8
Number of administered Lin^−^ cells (mean ± SD)		5.49 x 10^6^ ± 4.40
ALS-FRSr score (mean ± SD)	Before Lin^−^ Tx	29.17 ± 5.92
	3 days after Lin^−^ Tx	29.17 ± 5.92
	5 days after Lin^−^ Tx	29.17 ± 5.92
	7 days after Lin^−^ Tx	29.17 ± 5.92
	28 days after Lin^−^ Tx	29.75 ± 6.44
Norris scale score (mean ± SD)	Before Lin^−^ Tx	80.38 ± 17.49
	3 days after Lin^−^ Tx	82.83 ± 18.28
	5 days after Lin^−^ Tx	82.89 ± 18.34
	7 days after Lin^−^ Tx	82.89 ± 18.34
	28 days after Lin^−^ Tx	86.63 ± 17.30

## Supplementary Materials

Supplementary materials can be found at https://www.mdpi.com/1422-0067/21/3/1070/s1.

## Abbreviations

ALS	Amyotrophic lateral sclerosis
ALS-FRSr	Amyotrophic Lateral Sclerosis functional rating scale revised
BDNF	Brain-derived neurotrophic factor
CNS	Central nervous system
CRP	C-reactive protein
CSF	Cerebrospinal fluid
CXCR4	Chemokine receptor type 4
DEGs	Differentially expressed genes
FDA	Food and Drug Administration
GO	Gene ontology
Lin^−^	Lineage-negative
MNs	Motor neurons
NGF beta	Nerve growth factor beta
NT-3	Neurotrophin-3
NTs	Neurotrophins
PBS	Phosphate-buffered saline
sALS	Sporadic amyotrophic lateral sclerosis
SCs’	Stem cells
SOD1	Superoxide dismutase 1
SPCs	Stem and progenitor cells
Tx	Transplantation
