# Supplementary material for: Local and Systemic Humoral Response to Autologous Lineage-Negative Cells Intrathecal Administration in ALS Patients
Source: Int J Mol Sci. 2020 Feb 6;21(3):1070. doi: 10.3390/ijms21031070 (PMC7037134; doi:10.3390/ijms21031070)
Supplement: Supplementary file 1 [file ijms-21-01070-s001.zip › Supplementary File 3.pdf]

Supplementary File 3

List of 10 most up- and down-regulated genes for each individual patient in 7th day after cells administration.

Change in 7th day	Gene symbol	Gene name	Fold change
**Patient 1**			
Up	IGKV1-5	immunoglobulin kappa variable 1-5	5.50
Up	IGHG1	immunoglobulin heavy constant gamma 1 (G1m marker)	4.37
Up	SNORD18B	small nucleolar RNA, C/D box 18B	3.83
Up	SNORD55	small nucleolar RNA, C/D box 55	3.44
Up	IGHV3-23	immunoglobulin heavy variable 3-23	3.34
Up	HIST1H2AJ	histone cluster 1, H2aj	2.97
Up	IGHD	immunoglobulin heavy constant delta	2.92
Up	PI3	peptidase inhibitor 3, skin-derived	2.91
Up	SNORD23	small nucleolar RNA, C/D box 23	2.90
Up	C2CD4A	C2 calcium-dependent domain containing 4A	2.85
Down	HNRNPU-AS1	HNRNPU antisense RNA 1	-3.84
Down	GNAI3	guanine nucleotide binding protein (G protein), alpha inhibiting activity polypeptide 3	-3.91
Down	TAS2R30	taste receptor, type 2, member 30	-4.04
Down	TAF1D	TATA box binding protein (TBP)-associated factor, RNA polymerase I, D, 41kDa	-4.18
Down	ASAP1-IT2	ASAP1 intronic transcript 2 (non-protein coding)	-4.27
Down	ARHGAP15	Rho GTPase activating protein 15	-4.54
Down	DKFZp667F0711	uncharacterized protein DKFZp667F0711	-4.87
Down	PDE3B	phosphodiesterase 3B, cGMP-inhibited	-5.25
Down	ASAP1-IT1	ASAP1 intronic transcript 1 (non-protein coding)	-5.39
Down	TRAJ12	T cell receptor alpha joining 12	-5.40
**Patient 2**			
Up	HIST1H2AK	histone cluster 1, H2ak	12.81
Up	HIST1H2BF	histone cluster 1, H2bf	11.85
Up	SUMO2	small ubiquitin-like modifier 2	9.25
Up	MIR616	microRNA 616	9.09
Up	TRAJ35	T cell receptor alpha joining 35 (non-functional)	8.29
Up	CBWD1	COBW domain containing 1	8.06
Up	TRAJ34	T cell receptor alpha joining 34	7.72
Up	APOBEC3C	apolipoprotein B mRNA editing enzyme, catalytic polypeptide-like 3C	7.70
Up	SLCO4C1	solute carrier organic anion transporter family, member 4C1	7.34
Up	TRGJP1	T cell receptor gamma joining P1	7.26
Down	PP13	uncharacterized LOC100129503	-3.64
Down	PRAMEF26	PRAME family member 26	-3.69
Down	SNORA5C	small nucleolar RNA, H/ACA box 5C	-3.89
Down	RNU11	RNA, U11 small nuclear	-4.50
Down	GNB2L1	guanine nucleotide binding protein (G protein), beta polypeptide 2-like 1	-4.98
Down	RPS3A	ribosomal protein S3A	-5.09

Supplementary File 3

List of 10 most up- and down-regulated genes for each individual patient in 7th day after cells administration.

Down	SNORA61	small nucleolar RNA, H/ACA box 61	-5.25
Down	SNORA6	small nucleolar RNA, H/ACA box 6	-5.38
Down	SNORA72	small nucleolar RNA, H/ACA box 72	-7.42
Down	SNORA44	small nucleolar RNA, H/ACA box 44	-7.79
**Patient 3**			
**Up**	ZNF595	zinc finger protein 595	48.19
**Up**	USP17L5	ubiquitin specific peptidase 17-like family member 5	32.93
**Up**	USP17L15	ubiquitin specific peptidase 17-like family member 15	30.60
**Up**	SNORD115-5	small nucleolar RNA, C/D box 115-5	19.23
**Up**	SNORD115-20	small nucleolar RNA, C/D box 115-20	17.15
**Up**	DBET	D4Z4 binding element transcript (non-protein coding)	13.42
**Up**	SNORD115-4	small nucleolar RNA, C/D box 115-4	12.59
**Up**	SNORD115-44	small nucleolar RNA, C/D box 115-44	11.45
**Up**	DUX4L1	double homeobox 4 like 1	9.60
**Up**	DUX4	double homeobox 4	8.92
**Down**	SNORD102	small nucleolar RNA, C/D box 102	-14.82
**Down**	SNORA65	small nucleolar RNA, H/ACA box 65	-15.32
**Down**	SNORD34	small nucleolar RNA, C/D box 34	-17.16
**Down**	SNORD36C	small nucleolar RNA, C/D box 36C	-17.98
**Down**	SNORD56	small nucleolar RNA, C/D box 56	-19.58
**Down**	SNORA64	small nucleolar RNA, H/ACA box 64	-19.81
**Down**	SNORD16	small nucleolar RNA, C/D box 16	-24.26
**Down**	SNORA68	small nucleolar RNA, H/ACA box 68	-47.12
**Down**	SNORA72	small nucleolar RNA, H/ACA box 72	-53.82
**Down**	SNORA18	small nucleolar RNA, H/ACA box 18	-57.25
