# Supplementary material for: Local and Systemic Humoral Response to Autologous Lineage-Negative Cells Intrathecal Administration in ALS Patients
Source: Int J Mol Sci. 2020 Feb 6;21(3):1070. doi: 10.3390/ijms21031070 (PMC7037134; doi:10.3390/ijms21031070)
Supplement: Supplementary file 1 [file ijms-21-01070-s001.zip › Supplementary File 1.pdf]

Supplementary File 1

Correlations between plasma concentrations of analyzed factors and clinical outcome assessed using ALS-FRSr and Norris scale. r_s – Spearman's correlation coefficient. $p < 0.05$ is highlighted with bold font.

**Blood plasma concentrations**		**ALS-FRSr day 28 vs. 0** (valid calculations n = 16)		**Norris day 28 vs. 0** (valid calculations n = 16)	
		r_s	*p value*	*r_s*	*p value*
**CRP**	day 3 vs. 0	0.046	0.867	- 0.057	0.846
	day 5 vs. 0	0.042	0.876	0.195	0.505
	day 7 vs. 0	0.386	0.139	0.323	0.260
**BDNF**	day 3 vs. 0	- 0.218	0.417	- 0.110	0.708
	day 5 vs. 0	- 0.576	**0.019**	- 0.344	0.229
	day 7 vs. 0	- 0.383	0.143	- 0.131	0.656
**NGF beta**	day 3 vs. 0	- 0.116	0.668	- 0.082	0.779
	day 5 vs. 0	- 0.022	0.935	0.037	0.901
	day 7 vs. 0	- 0.217	0.420	0.174	0.552
**NT-3**	day 3 vs. 0	- 0.278	0.296	0.010	0.972
	day 5 vs. 0	- 0.218	0.417	0.200	0.493
	day 7 vs. 0	0.006	0.982	0.338	0.237
