# Supplementary material for: Local and Systemic Humoral Response to Autologous Lineage-Negative Cells Intrathecal Administration in ALS Patients
Source: Int J Mol Sci. 2020 Feb 6;21(3):1070. doi: 10.3390/ijms21031070 (PMC7037134; doi:10.3390/ijms21031070)
Supplement: Supplementary file 1 [file ijms-21-01070-s001.zip › Supplementary File 2.pdf]

Supplementary File 2

Detailed characteristic of three patients randomly chosen for microarray gene expression analysis.

	Patient 1	Patient 2	Patient 3
**Age (years)**	61	60	55
**Sex**	Male	Male	Female
**ALS duration (months)**	96	25	36
**Number of obtained bone marrow mononuclear cells (x10^6)**	140	192.5	150
**Number of administered Lin^- cells (x10^6)**	2.0	1.8	2.3
**ALS-FRSr result in Day 0**	29	29	22
**ALS-FRSr result in 28th day**	34	24	22
**Norris scale result in Day 0**	79	80	75
**Norris scale result in 28th day**	100	77	75
